# Synergistic Effect of Plant Extracts on Endodontic Pathogens Isolated from Teeth with Root Canal Treatment Failure: An In Vitro Study

**DOI:** 10.3390/antibiotics10050552

**Published:** 2021-05-09

**Authors:** Suraj Arora, Shahabe Abullais Saquib, Youssef A Algarni, Mohammed Abdul Kader, Irfan Ahmad, Mohammad Y Alshahrani, Priyanka Saluja, Suheel Manzoor Baba, Anshad M. Abdulla, Shashit Shetty Bavabeedu

**Affiliations:** 1 Department of Restorative Dental Sciences, College of Dentistry, King Khalid University, Abha 61321, Saudi Arabia; yalgarni@kku.edu.sa (Y.AA.); msaheb@kku.edu.sa (M.A.K.); baba@kku.edu.sa (S.M.B.); sbavabeedu@kku.edu.sa (S.S.B.); 2 Department of Periodontics and Community Dental Sciences, College of Dentistry, King Khalid University, Abha 61321, Saudi Arabia; sshahabe@kku.edu.sa; 3 Department of Clinical Laboratory Sciences, College of Applied Medical Sciences, King Khalid University, Abha 61321, Saudi Arabia; imahmood@kku.edu.sa (I.A.); moyahya@kku.edu.sa (M.YA.); 4 Department of Conservative Dentistry and Endodontics, JCD Dental College, Sirsa 125055, India; priyanka.salujaarora@gmail.com; 5 Department of Pediatric Dentistry & Orthodontics, College of Dentistry, King Khalid University, Abha 61321, Saudi Arabia; anshad@kku.edu.sa

**Keywords:** endodontic pathogens, synergy, plant extracts, root canal treatment failure, antimicrobial activity

## Abstract

Background and objectives: This study aimed to evaluate the synergistic antimicrobial activity of extracts obtained from *Salvadora persica* (Miswak), *Commiphora molmol* (myrrh) and *Azadirachta indica* (neem) in combination with commercially available antimicrobial agents: penicillin, tetracycline, ofloxacin and fluconazole on endodontic pathogens such as *Enterococcus faecalis, Streptococcus mitis,* *Actinomyces naeslundii* and *Candida albicans*. Materials and Methods: Microbiological samples from the root canals of the teeth undergoing retreatment were taken using sterile paper points kept at full length in the canal for 30 s. The disc diffusion method was used to check the susceptibility of microbes to the plant extracts and antimicrobials by measuring the diameter of the inhibition zones. Against the microbes, minimum inhibitory concentration (MIC) and minimum bactericidal concentration (MBC)/minimum fungicidal concentration (MFC) of the plant extracts were assessed. The fractional inhibitory concentration index (FICI) was used to estimate the synergistic effect of plant extracts combined with antimicrobials against the resistant endodontic microbes. Results: The findings clearly indicate the effectiveness of all the three plant extracts, *Commiphora molmol, Azadirachta indica, Salvadora persica,* against all the experimental pathogenic microorganisms except for the ineffectiveness of *Azadirachta indica, Salvadora persica* against *Candida* *albicans.* Maximum antimicrobial activity was displayed by *Azadirachta indica* against *Enterococcus* *faecalis* (MIC = 0.09 ± 1.2 mg/mL, MBC = 0.78 ± 1.25 mg/mL) and the minimum antimicrobial activity was displayed by *Commiphora molmol* against *Actinomyces naeslundii* (MIC = 12.5 ± 3.25 mg/mL, MBC = 100 ± 3.75 mg/mL). The best synergy was displayed by *Commiphora molmol* with fluconazole against *Candida* *albicans* (FICI = 0.45). Conclusions: The current study delineates the variable antimicrobial activity of plant extracts against the experimental endodontic pathogenic microorganisms. Plant extracts in conjunction with various antimicrobials can be valuable aids in combating relatively resistant endodontic microorganisms that have been the cause of worry in recent years, leading to failure even in treatment procedures following all required protocols.

## 1. Introduction

*Enterococci* are the most common bacteria found in the oral cavity, gastrointestinal tract, and vagina of humans and animals. Previously, *Enterococci* were regarded as non-virulent, but now they are recognized as one of the major causes of nosocomial infections worldwide [1]. *Enterococci* are Gram-positive cocci that can occur singly, in pairs, or as short chains. They are facultative anaerobes, possessing the ability to grow in the presence or absence of oxygen. In endodontics, *Enterococcus* species, especially *Enterococcus faecalis* (*E. faecalis*), are associated with chronic periodontitis and failed root canal treatments involving chronic apical periodontitis [2,3].

*Enterococcus* pose a great challenge for dentists owing to their complex nature, which helps them to survive the harsh environments, including extreme alkaline pH, salt concentrations and high temperatures of 60 °C [4,5,6]. The principal cause for *E. faecalis* to be associated with endodontic failure is its ability to invade dentinal tubules and strong adhesion to collagen [7], which is abundantly present in root dentin and cementum. A confocal laser-scanning microscope showed that the depth of viable *E. faecalis* ranges from 100 to 400 μm into dentinal tubules.

The virulence traits of *E. faecalis* are cell surface-associated protein, namely, enterococcal surface protein (ESP), secreted toxins, such as cytolysin, hemolysin, gelatinase, aggregation substance (AS), serine protease, and cell wall polysaccharide. These virulence traits are attributed to the pathogenicity islands, which are virulence-coding genes present on the genome. These genes encode for transposases, transcriptional regulators, and proteins are known to have potential roles in enhancing virulence [8]. *E. faecalis* has begun to pose a therapeutic challenge to physicians due to its ease of acquiring and transferring antimicrobial drug resistance. Management of such cases can sometimes be more demanding with the complex internal root canal anatomy of some teeth. However, this aspect has been greatly solved with the advent of cone-beam computed tomography (CBCT) in the field of endodontics [9,10,11].

*Candida albicans (C. albicans)* is a fungus usually seen in 21% of primary infections and 18% of retreatment cases [12,13]. *Candida* can survive in extreme environments by biofilm formation and using its physiochemical properties to suit the local conditions. This is the prime reason that it can persist in highly alkaline environments created by calcium hydroxide medications [14]. *Actinomyces naeslundii (A. naeslundii)* and other actinomyces species cause extra radicular infections [13]. While most extra-radicular infections are a sequel to intra-radicular ones, apical *Actinomycosis*, caused by *Actinomyces* species, is an example of extra-radicular infection independent of the intra-radicular infections [15]. This microorganism probably migrates from periapical tissues to infect the root canal system, but how this bacterium invades the periapical tissues is still controversial. It may result from not following the root canal treatment procedure in the recommended way leading to sepsis. *Streptococcus mitis (S. mitis)* is a Gram-positive coccus normally found in the oral cavity and can attach tooth surfaces by expressing specific proteins known as adhesins. Its cell wall contains peptidoglycans and lipoteichoic acids, which can influence inflammatory reactions and enhance pain modulation [16]. *Streptococcus* can invade dentinal tubules and, by releasing different extracellular proteins, can adapt and survive in adverse environmental conditions [17].

*Salvadora persica* (*S. persica*)*,* also known as miswak, has antimicrobial properties and prevents *E. faecalis* bacteria from attaching the tooth surface [18]. It has also been shown to reduce *Streptococcus mutans* (S. *mutans)* count as fluoride interacts with bacterial glycolytic enzymes [19]. Because of its good antimicrobial properties [20] and low cytotoxicity can be used as a root canal irrigant as an alternative to sodium hypochlorite. Myrrh is a resin acquired as an exudate from the trunk of *Commiphora molmol (C. molmol)*. Myrrh belongs to the family Burseraceae [21], which are trees that grow in sand and rocky areas in several countries like Saudi Arabia, Somalia, Sudan, Yemen, and Northeast Africa. *C. molmol* consists of 57–61% water-soluble gum, 25–40% alcohol-soluble resin, 7–17% volatile oil, and 3–4% impurities. In ancient times, it was used in inflammatory and infectious diseases. *Azadirachta indica* (*A. indica*), commonly known as neem, is a tree, which belongs to the Meliaceae family. It has many biological activities, such as antimicrobial, antiviral, antifungal, anti-inflammatory, antimalarial, antipyretic, antioxidant, analgesic, immune-stimulant, anti-fertility, anti-acne, antihypoglycemic, anti-cancer and nematicidal properties [22,23].

This study was aimed to evaluate the synergistic antimicrobial activity of extracts obtained from *S. persica*, *C. molmol* and *A. indica* combined with commercially available antimicrobial agents: penicillin, tetracycline, ofloxacin and fluconazole on endodontic pathogens, such as *E. faecalis, S. mitis, A. naeslundii and C. albicans*.

## 2. Materials and Methods

### 2.1. Study Design and Protocol

An in vitro experimental study design was used for the current study. Patients visiting the Department of Endodontics, College of Dentistry, King Khalid University, Saudi Arabia for retreatment of failed root canals from May 2018 to May 2019 were included in the study. The patients having radiological proof depicting apical periodontitis following diagnostic directions accepted by AAE [24] were included in this study. Patients with a history of smoking, periodontal pockets, pregnant women, and those having systemic conditions and taking antimicrobial drugs were excluded. Additionally, patients with missed canals, instrument separation, calcified canals and perforations were also excluded. Ethical approval (SRC/ETH/2017–18/085) was taken from the Research Ethics Committee, College of Dentistry, King Khalid University, Saudi Arabia, and written informed consent was obtained from the patients interested in voluntary participation.

**Figure 1:**
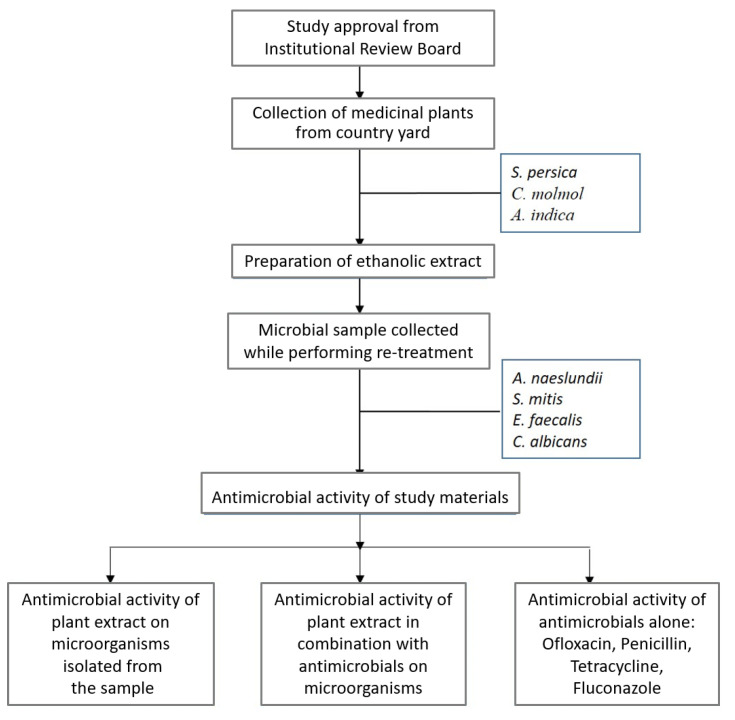
Flowchart of study design.

Raw herbal products (roots and stems of *S. persica*, gum resin of *C. molmol* and bark of *A. indica*) were taken from the traditional market of Abha, Saudi Arabia. The reliability of the specimens was checked by the plant taxonomist. Various antimicrobials, such as penicillin, tetracycline, ofloxacin and fluconazole (Sigma-Aldrich, Merck KGaA, Darmstadt; Germany) in blend with various plant extracts were used to assess the presence of the collaborative effect. The detailed protocol of the study is depicted in the flowchart (Figure 1).

### 2.2. Plants Extract Preparation

The dried herbs of *S. persica, C. molmol and A. indica* were powdered using a grinder. Muslin cloth was used to pack 50 g of the powder and treated in a Soxhlet extractor using absolute ethanol for 72 h to perform hot extraction. Additionally, a muslin cloth was used to filter the ethanolic extract later by Whatman 1 filter paper. A rotary evaporator (Buchi Rotavapor R-200) was used to vaporize the filtrate underneath decreased pressure and temperature. In ethanol (0.2 g/mL), dried plant extracts were dissolved again to be used in antimicrobial susceptibility assay. Preparation of stock solutions was done, and two-fold dilution of the stock ranging from 50 μg/mL to 0.2g/mL was used to achieve a final working volume to be later used in determining MIC and MBC/MFC.

### 2.3. Clinical Examination and Sample Collection

The teeth undergoing retreatment were assessed for their location, number of canals, and presenting signs and symptoms (pain, pus drainage through sinus tract, mobility and periodontal status). Intracoronal and extracoronal restorations were considered unacceptable if they had a marginal defect, fracture, secondary caries, or a dislodged restoration. A periapical radiograph was used to evaluate the condition of obturation [25]. An obturation was regarded as unacceptable if: (i) the obturation was more than 2 mm short of the apex or overextended beyond the apex; (ii) lateral space or voids were there in the obturation. Two investigators were assigned for the clinical evaluation. Samples from the root canals were taken using the procedure reported by Gomes et al. [16]. After rubber dam application, the concerned tooth was cleaned with 5.25% sodium hypochlorite, followed by 5% sodium thiosulfate. After removing the old restoration, the root canals were located, and the pulp chamber was cleaned with 5.25% sodium hypochlorite. The old obturation material was removed from the canals using H files (Dentsply Tulsa Dental, Tulsa, OK, USA) with sterile saline as an irrigant, and canal patency was obtained. Sterile paper points were used to take the microbial samples at full length in the canal for 30 s. These paper points were then kept in a tube containing 1.5 mL of Gotenberg agar III as the transport medium.

### 2.4. Media for Microbial Growth

Microbial growth and isolation were done in the specific culture media presented in Table 1. The microbial strains were further incubated at 37 °C for 24–48 h. The desired microbes grown on the media were further identified based on the colony morphology; biochemical tests were used to identify species.

### 2.5. Biochemical Tests for Microbial Identification

An *E. faecalis* colony was isolated for identification by biochemical tests, such as Voges–Prosjauer, indole, citrate, urease, hydrogen sulfide (H2S) production and L-pyrrolidinyl arylamidase (PYR) test. *A. neslundii* and *S. mitis* were tested by API rapid ID32A kit (bioMérieux) as described by the manufacturer. They were also tested for esculin hydrolysis. Acid production from arabinose, fructose, cellobiose, inositol, glycogen, mannitol, lactose, ribose, and trehalose (at 1% *w*/*v*) and salicin and starch (at 0.5% *w*/*v*) in peptone-yeast extract broth was observed. *C. albicans* colonies showed a rough, filamentous border, composed of hyphae and pseudohyphae. *C. albicans* was identified by a germ tube test.

### 2.6. Microbiological Assay

#### 2.6.1. Antimicrobial Susceptibility Assays of the Antimicrobial Agents

Disc diffusion method was used to examine antimicrobial sensitivity assay. Lysogeny broth (LB) media were used to inoculate cultures of microbes at 37 °C for 3 h. Phosphate-buffered saline was used to adjust turbidity to 0.5 McFarland’s index. Mueller–Hinton agar (MHA) plates were used for microbial lawn culture. MHA plates were used to place the selected antimicrobial plates (penicillin, tetracycline, ofloxacin and fluconazole) and incubated in suitable conditions at 37 °C for 24 h. The microbial growth inhibition zone was measured around each antimicrobial agent in millimeters as described [26].

#### 2.6.2. Antimicrobial Susceptibility Assays of the Plant Extracts

The agar well diffusion method was used to examine the antimicrobial activity of the extracts. LB broth media was inoculated with cultures of microbes for 3 h at 37 °C, and 0.5 McFarland’s index in phosphate-buffered saline was used to adjust the turbidity. The cap of the sterile syringe was used to prepare the wells of 6 mm diameter in LB agar, and a lawn culture was formed from a diluted culture with the help of a sterile cotton swab. The wells were filled with 20 μL of extract from various plants (0.2 g/mL) with incubation of the plates at 37 °C for 24 h. The diameter of the inhibition zone was measured around each well in millimeters as described [26]. The well for each microbe having 20 μL of ethanol without any extracts was deliberated as control. The inhibition zone produced by plant extracts was subtracted from the inhibition zone produced by ethanol.

#### 2.6.3. Determination of MIC

Micro-broth dilution assays using Mueller–Hinton broth were used to determine MICs of the plant extracts against microbial strains. The range of the extract’s concentration was from 50 μg/mL to 0.2 g/mL. The wells of polystyrene sterile flat-bottomed 96-well plates were filled with 180 μL culture of all microbial strains. The triplicate wells for each strain were loaded with 20 μL from the 2-fold dilution of the plant extract. In the control group, the triplicate wells were loaded with 20 μL of ethanol (5%). Each strain had a starting inoculum of 1.5 × 10^5^ CFU/mL. MIC was considered as the lowest concentration of extracts that showed no visible microbial growth and turbidity after 24 h of incubation.

#### 2.6.4. Determination of MBC/MFC

Subculturing of 100 μL of the culture from each well of the micro-broth assay was done on MHA plates at 37 °C for 24 h to obtain the MBC/MFC of the extracts. MBC/MFC was considered as the lowest concentration of the extracts at which there was no microbial growth.

#### 2.6.5. Synergistic Antimicrobial Assays

The synergistic antimicrobial activity of the plant extracts was determined in combination with various antimicrobial agents by checkboard methods.

The antimicrobial activity of the plant extract and antimicrobial agent combination was interpreted as one of the following categories: synergy; additive effect, indifferent; or antagonism. The fractional inhibitory concentration (FIC) of each agent was calculated as the MIC of the agent in combination, divided by the MIC of the agent alone.

$$\mathrm{FIC}\;\left(a\right)=\frac{\mathrm{MIC}\;\left(a\right)\;\mathrm{in}\;\mathrm{combination}\;\mathrm{with}\;\left(b\right)}{\mathrm{MIC}\;\left(a\right)\;\mathrm{alone}}$$

$$\mathrm{FIC}\;\left(b\right)=\frac{\mathrm{MIC}\;\left(b\right)\;\mathrm{in}\;\mathrm{combination}\;\mathrm{with}\;\left(a\right)}{\mathrm{MIC}\;\left(b\right)\;\mathrm{alone}}$$

The sum of the FIC or FIC index (FICI) is therefore, analyzed as:

ΣFIC = FIC (a) + FIC (b)

The FICI results were interpreted as follows: <0.5 synergy; 0.5 to 1 additive effect; 1–2 indifferent or no effect; and >2 antagonism [27].

### 2.7. Statistical Analysis

The experiments were conducted in triplicates. For the results, data were outlined as mean ± standard deviation (SD). SPSS (version 11.5, Chicago) was used for statistical analysis. The significance of the difference between the mean expression of the experimental and the control samples was calculated using a one-tail Student’s *t*-test.

## 3. Results

### 3.1. Antimicrobial Activity of Antimicrobial Agents and Plant Extracts

In this study, the screening activity of the antimicrobials against different strains was performed before evaluating the antimicrobial activity of the extracts from various plants. Table 2 and Table 3 presented the outcomes of antimicrobial sensitivity of the various microbes to the antimicrobials used. During the experimental period, all the microbes were resistant to tetracycline. *S. mitis* was resistant to all the antimicrobials.

**Figure 2:**
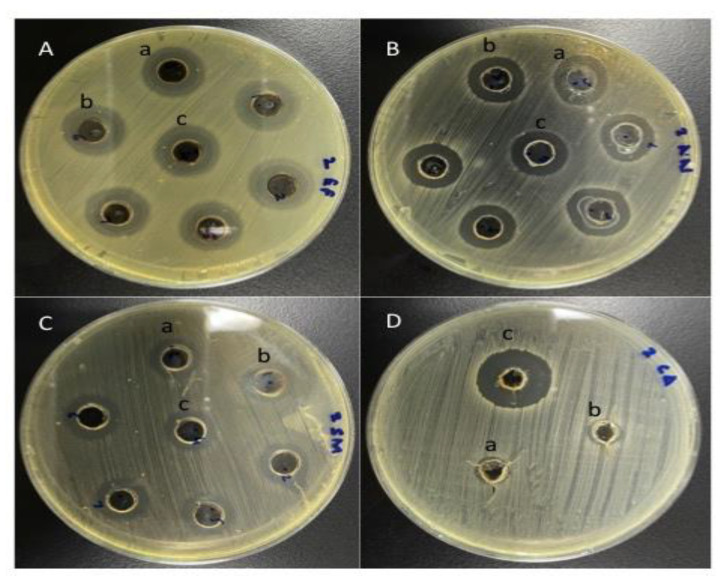
Antimicrobial activity of plant extracts *A. indica*, *S. persica* and *C. molmol* against the microbial strains (**A**) *E. faecalis* (**B**) *A. neslundii* (**C**) *S. mitis* (**D**) *C. albicans*.

To assess antimicrobial activity, the various plant extracts were used to treat all four microbes. A zone of inhibition was viewed as significant for susceptibility of the microbe to the plant extract if the zone size was more than 8 mm. Table 4 presents the antimicrobial activity, MIC and MBC/MFC shown by various plant extracts against the tested microbes. All tested microbial strains were susceptible to ethanolic extracts of myrrh, while neem and miswak were found effective against all the tested microbes except *C. albicans* (Figure 2) (*p* < 0.05).

### 3.2. MIC and MBC/MFC of Plant Extracts

To compare the effects of various plant extracts on microbial growth, MIC and MBC/MFC of these extracts were considered. Since the diffusion and absorption of extract-bioactive compounds may curb the effects on microbial proliferation, they were not considered in the method of liquid dilution. It was found that all the microbial strains were susceptible to myrrh (*p* < 0.05), while *C. albicans* was not susceptible to neem and miswak extract (*p* = 0.179). The highest MIC and MBC value was displayed by *C. molmol* against *A. neslundii,* whereas the lowest MIC and MBC value was displayed by *A. indica* against *E.* *faecalis* (Table 4).

### 3.3. Synergistic Activity of Plant Extracts with Antimicrobial Agents

The best effect of *A. indica* was against *E. faecalis,* where it showed additive effect when it was combined with penicillin, tetracycline and ofloxacin. It also exhibited an additive effect with ofloxacin against *S. mitis*. Combination of *A. indica* with penicillin, tetracycline and ofloxacin showed no effect against *A. neslundii.* (Table 5).

The combination of *S. persica* with penicillin showed an additive effect against *E. faecalis,* while it showed an additive effect against *A. neslundii* when it was combined with tetracycline and ofloxacin. No effect of *S. persica* was observed against *E. faecalis* and *A. neslundii* when it combined with tetracycline, ofloxacin and penicillin, respectively. In the case of *S. mitis,* when *S. persica* was combined with ofloxacin, it exhibited a synergistic effect. (Table 6).

*C. molmol* had a synergy with ofloxacin against *S. mitis* and with fluconazole against *C. albicans*. It showed an additive effect against *E. faecalis* in combination with penicillin, tetracycline and ofloxacin. At the same time, it exhibited no effect in combination of penicillin and ofloxacin against *A. neslundii* (Table 7).

## 4. Discussion

In the last 15 years, the role of stem cells has given a major boost to various dental regenerative procedures for repairing dental and other oral defects [28,29,30]. However, all these procedures require aseptic conditions, which is greatly altered by the presence of pathogenic oral microorganisms. The study was conducted on *E. faecalis, S. mitis, A, neslundii,* and *C. Albicans* as these microorganisms are commonly responsible for the failure of the root canal treatment procedure and result in persistent post-treatment apical periodontitis [15,16,31,32,33,34,35].

The aim of this in vitro study was to investigate synergies between plant extracts of *C. molmol, A. indica* and *S. persica* with various antimicrobials against endodontic pathogenic microorganisms, such as *E. faecalis, S. mitis, A. neslundii* and *C. albicans*. *E. faecalis* can be found as the only microorganism in the root canal treated teeth with periapical pathology [34,36]. Its frequency is higher in persistent peri-radicular infections and almost nine times in failed root canals than primary endodontic infections [37]. In *C. albicans,* dimorphic switching plays an important role in its pathogenicity and biofilm formation [38,39].

Ethanol was used as a solvent to withdraw the vital compounds from the plant products following the previous studies [40,41,42]. The existence of saponins, steroids, tannic acid, alkaloids, flavonoids, glycosides and anthraquinone renders antimicrobial properties of *A. indica* (neem) [43]. Several studies have demonstrated the antimicrobial effect of *A. indica* on *E. faecalis* [44,45,46]. An in vitro study found that the antimicrobial effect of *A. indica* against *C. albicans* followed sodium hypochlorite and propolis [47]. On the contrary, in the present study, *A. indica* demonstrated antimicrobial property against all endodontic pathogenic microbes (*p* < 0.05) except the *C. albicans* (*p* = 0.179). The antibacterial activity of neem was the most against *E. faecalis* (MIC=0.09 ± 1.2 mg/mL, MBC=0.78 ± 1.25 mg/mL) followed by *A. neslundii* (MIC=4 ± 0.5 mg/mL, MBC=16 ± 0.65 mg/mL) and *S. mitis* (MIC=6.25 ± 0.24 mg/mL, MBC=100 ± 2.5 mg/mL).

Miswak in the form of chewing sticks, due to their antibacterial, antifungal, antiviral, and antiplaque properties, have a widespread application in and around Middle Eastern countries [20]. Against oral pathogens, it has effectively reduced the count of *E. faecalis, S. mutans, L. acidophilus* and *P. aeruginosa* [48,49]. In the present study, Miswak showed antimicrobial properties against all the pathogens other than *C. albicans.* The highest antibacterial activity of miswak was displayed against *S. mitis* (MIC= 6.25 ± 1.5 mg/mL, MBC = 50 ± 1.5) followed by *E. faecalis* (MIC = 6.25 ± 2.25 mg/mL, MBC = 100 ± 1.75 mg/mL) and *A. neslundii* (MIC = 8.65 ± 0.50 mg/mL, MBC = 12.34 ± 1.0 mg/mL).

Myrrh has been investigated for its antimicrobial properties against oral pathogens and found to have promising results against *E. faecalis*, *F. nucleatum* [50] and helping in the reduction of plaque and gingival inflammation. In the current study, myrrh was the only plant extract that was an effective antimicrobial against all the experimental pathogens. It displayed the highest antimicrobial effect against *C. albicans* (MIC = 3.12± 0.75 mg/mL, MFC = 25 ± 1.5 mg/mL) followed by *S. mitis* (MIC = 3.25 ± 2.25 mg/mL, MBC = 50 ± 2.25 mg/mL), *E. faecalis* (MIC = 6.25 ± 1.50 mg/mL, MBC = 100 ± 2.0 mg/mL) and *A. neslundii* (MIC = 12.5 ± 3.25 mg/mL, MBC = 100 ± 3.75 mg/mL).

The effect of combining two drugs can be antagonistic, indifferent, additive, or synergistic. Few previous in vitro studies have evaluated the effects of combining various antibiotics with different plant extracts that have reduced MIC for antibiotics [51,52]. One of the important aspects of the current study was to evaluate the effect of combining the various antimicrobial drugs with different plant extracts on endodontic pathogens responsible for root canal failure, which may be helpful in the development of a better and new protocol for treating complex endodontic cases. Neem had displayed an additive effect with penicillin (FICI = 0.83), tetracycline (FICI = 0.96), ofloxacin (FICI = 0.88) against *E. faecalis,* while for *A. neslundii* it was indifferent with penicillin (FICI = 1.23), tetracycline (FICI = 1.18) and ofloxacin (FICI = 1.005). The best effect of combining various plant extracts with different antimicrobials against *E. faecalis* was an additive effect of combining *C. molmol* with penicillin (FICI = 0.51), which was followed by *C. molmol* with tetracycline (FICI = 0.57) and *C. molmol* with ofloxacin (FICI = 0.71). Miswak was effective against *S. mitis* in synergy with ofloxacin (FICI = 0.49). Against *A. neslundii,* it had an additive effect with tetracycline (FICI = 0.64) and ofloxacin (FICI = 0.91), and against *E. faecalis,* it had an additive effect with penicillin (FICI = 0.79). The best effect of combining various plant extracts with different antimicrobials against *A. neslundii* was an additive effect of combining *C. molmol* with tetracycline (FICI =0.54 ), which was followed by *S. persica* with tetracycline (FICI = 0.64) and *S. persica* with ofloxacin (FICI = 0.91). Myrrh was in synergy with ofloxacin and fluconazole against both *S. mitis* (FICI = 0.49) and *C. albicans* (FICI = 0.45), respectively. It exhibited an additive effect against *E. faecalis* with all the three antimicrobials: penicillin (FICI = 0.51), tetracycline (FICI = 0.57), ofloxacin (FICI = 0.71), while an indifferent effect was observed against *A. neslundii* with penicillin (FICI = 1.25) and ofloxacin (FICI = 1.40). The best effects of combining various plant extracts with different antimicrobials against *S. mitis* were the synergistic effect of *C. molmol* with ofloxacin (FICI = 0.49) and *S. persica* with ofloxacin (FICI = 0.49). Against *C. albicans,* the synergistic effect of *C. molmol* with fluconazole, was the best synergistic effect (FICI = 0.45) against the different microbes tested with various combinations.

Tetracyclines, being broad-spectrum antibiotics, are used as intracanal antibiotics against polymicrobial endodontic microorganisms, along with others like metronidazole and newer BioPure MTAD (Dentsply Sirona, Salzburg, Austria). However, intracanal microorganisms are resistant to these antibiotics [53,54,55], and in some cases, tetracycline may even promote the growth of *C. albicans* [56]. This problem of antimicrobial resistance can be effectively managed by using the combination of plant extracts with antimicrobial agents. Along with this, they can be used alone for intracanal irrigation as an alternative to sodium hypochlorite, which has many disadvantages like toxicity, bad taste, and accident mishaps. Further, more evidence gained with future studies combining these plant extracts with antimicrobials may help with other dental procedures like pulp capping, regenerative endodontics and managing traumatic injuries like luxation and tooth avulsion.

## 5. Conclusions

The results from the study indicate that plants in the form of their extracts are good sources of biologically active antimicrobial agents. Plant extracts in conjunction with various antimicrobial agents can be a valuable aid in combating the relatively resistant endodontic microorganisms that have been the cause of worry in recent years leading to failure even in the treatment procedures following all the required protocols. The current study clearly indicates the effectiveness of all the three plant extracts, *C. molmol, A. indica, S. persica,* against all the experimental endodontic pathogenic microorganisms. *A. indica and S. persica* were ineffective against *C. albicans*. Synergies of plant extracts with antimicrobials helped enhance overall antimicrobial effects. The best synergy was displayed by *C. molmol* with fluconazole against *C.* *albicans.* In addition, *C. molmol and S. persica* both exhibited synergy with ofloxacin against *S. mitis.* The effects of synergies may help in improving the treatment modalities against endodontic pathogens. However, further studies are needed to evaluate their biocompatibility, effect on biofilms and their mechanisms for synergy.

## Figures and Tables

**Table 1:** Selective media used.

Organisms	Media
***E. faecalis***	Enterococcosel agar (BBL Microbiological Systems, Cockeysville, MD, USA)
***A. neslundii***	Brain heart infusion agar (BHI agar, HiMedia, Mumbai, India) supplemented with 10% defibrinated sheep blood
***S. mitis***	Brain heart infusion agar/broth
***C. albicans***	Blood agar (Merck, Germany)

**Table 2:** Antimicrobial activity, MIC and MBC exhibited by antimicrobials against endodontic pathogenic microorganisms.

Organisms	Penicillin (10 µg)			Tetracycline (30 µg)			Ofloxacin (5 µg)		
	Zone (mm) Mean ± SD	MIC (µg/mL) Mean ± SD	MBC (µg/mL) Mean ± SD	Zone (mm) Mean ± SD	MIC (µg/mL) Mean ± SD	MBC (µg/mL) Mean ± SD	Zone (mm) Mean ± SD	MIC (µg/mL) Mean ± SD	MBC (µg/mL) Mean ± SD
***E. faecalis***	15 ± 2.65 (S)	5.5 ± 1.5	12 ± 2.25	0 (R)	8 ± 0.75	14.25 ± 1.35	17 ± 2.65 (S)	6 ± 1.65	10.28 ± 3.25
***A. neslundii***	13 ± 1.5 (S)	2 ± 1.5	4.3 ± 0.69	3 ± 0.5 (R)	2 ± 1.45	4.34 ± 0.85	15 ± 1.5 (S)	2 ± 0.75	5.5 ± 1.65
***S. mitis***	0 (R)	-	-	0 (R)	-	-	2 ± 0.75 (R)	1.9 ± 1.35	4.3 ± 3.47

MIC = minimum inhibitory concentration; MBC = minimum bactericidal concentration; S = sensitivity; R = resistance; SD = standard deviation; - = no activity at the concentration of the antimicrobials used.

**Table 3:** Antifungal activity, MIC and MFC exhibited by fluconazole against *C. albicans*.

Organisms	Fluconazole (10 µg)		
	Zone (mm) Mean ± SD	MIC (µg/mL) Mean ± SD	MFC (µg/mL) Mean ± SD
***C. albicans***	24 ± 2.75 (S)	1 ± 0.05	2.15 ± 0.35

MIC = minimum inhibitory concentration; MFC = minimum fungicidal concentration; S = sensitivity; SD = standard deviation.

**Table 4:** Antimicrobial activity, MIC and MBC/MFC exhibited by plant extract against endodontic pathogenic microorganisms.

Organisms	*A. indica*			*S. persica*			*C. molmol*			
	Zone (mm) Mean ± SD	MIC (mg/mL) Mean ± SD	MBC/MFC (mg/mL) Mean ± SD	Zone (mm) Mean ± SD	MIC (mg/mL) Mean ± SD	MBC/MFC (mg/mL) Mean ± SD	Zone (mm) Mean ± SD	MIC (mg/mL) Mean ± SD	MBC/MFC (mg/mL) Mean ± SD	*p*-Value
***E. faecalis***	14 ± 1.5	0.09 ± 1.2	0.78 ± 1.25	13 ± 1.75	6.25 ± 2.25	100 ± 1.75	17 ± 1.25	6.25 ± 1.50	100 ± 2.0	0.038
***A. neslundii***	9 ± 1.0	4 ± 0.5	16 ± 0.65	20 ± 2.0	8.65 ± 0.50	12.34 ± 1.0	13 ± 0.75	12.5 ± 3.25	100 ± 3.75	0.013
***S. mitis***	10 ± 0.75	6.25 ± 0.24	100 ± 2.5	12 ± 1.25	6.25 ± 1.5	50 ± 1.5	15 ± 2.75	3.25 ± 2.25	50 ± 2.25	0.031
***C. albicans***	-	-	-	-	-	-	14 ± 0.68	3.12 ± 0.75	25 ± 1.5	0.179

MIC = minimum inhibitory concentration; MBC = minimum bactericidal concentration; MFC=minimum fungicidal concentration; - = no activity at the concentration of the extracts used; SD=standard deviation.

**Table 5:** Synergistic antimicrobial activity of *A. indica* with various antimicrobial agents.

Organisms	Antibiotics	MIC of Antimicrobial Agents (μg/ mL)		MIC of *A. indica* (mg/mL)		FICI	Interpretation
		Alone	Combination	Alone	Combination		
***E. faecalis***	Penicillin	5.5	2.5	0.9	0.035	0.83	Additive
	Tetracycline	8	4	0.9	0.042	0.96	Additive
	Ofloxacin	6	3.75	0.9	0.06	0.88	Additive
***A. naeslundii***	Penicillin	2	1.5	4	1.95	1.23	Indifferent
	Tetracycline	2	0.75	4	3.25	1.18	Indifferent
	Ofloxacin	2	1.25	4	1.55	1.005	Indifferent
***S. mitis***	Penicillin	−	−	−	−	−	−
	Tetracycline	−	−	−	−	−	−
	Ofloxacin	1.9	0.65	6.25	1.75	0.62	Additive

MIC = minimum inhibitory concentration; FICI = fractional inhibitory concentration index; - = no activity.

**Table 6:** Synergistic antimicrobial activity of *s. persica* with various antimicrobial agents.

Organisms	Antibiotics	MIC of Antimicrobial Agents (μg/ mL)		MIC of *S. persica* (mg/mL)		FICI	Interpretation
		Alone	Combination	Alone	Combination		
***E. faecalis***	Penicillin	5.5	2.5	6.25	2.15	0.79	Additive
	Tetracycline	8	4	6.25	4.25	1.18	Indifferent
	Ofloxacin	6	3.75	6.25	3.5	1.185	Indifferent
***A. naeslundii***	Penicillin	2	1.5	8.65	8.65	1.75	Indifferent
	Tetracycline	2	0.75	8.65	2.35	0.64	Additive
	Ofloxacin	2	1.25	8.65	2.5	0.91	Additive
***S. mitis***	Penicillin	−	−	−	−	−	−
	Tetracycline	−	−	−	−	−	−
	Ofloxacin	1.9	0.65	6.25	0.95	0.49	Synergistic

MIC = minimum inhibitory concentration; FICI = fractional inhibitory concentration index; - = no activity.

**Table 7:** Synergistic antimicrobial activity of *C. molmol* with various antimicrobial agents.

Organisms	Antibiotics	MIC of Antimicrobial Agents (μg/ mL)		MIC of *C. molmol* (mg/mL)		FICI	Interpretation
		Alone	Combination	Alone	Combination		
***E. faecalis***	Penicillin	5.5	2.5	6.25	0.40	0.51	Additive
	Tetracycline	8	4	6.25	0.45	0.57	Additive
	Ofloxacin	6	3.75	6.25	0.58	0.71	Additive
***A. naeslundii***	Penicillin	2	1.5	12.5	0.25	1.25	Indifferent
	Tetracycline	2	0.75	12.5	2.21	0.54	Additive
	Ofloxacin	2	1.25	12.5	9.75	1.40	Indifferent
***S. mitis***	Penicillin	−	−	−	−	−	−
	Tetracycline	−	−	−	−	−	−
	Ofloxacin	1.9	0.65	3.25	0.5	0.49	Synergistic
***C. albicans***	Fluconazole	1	0.31	3.12	0.44	0.45	Synergistic

MIC = minimum inhibitory concentration; FICI = fractional inhibitory concentration index; - = no activity.

## Data Availability

The data presented in this study are available on reasonable request from the corresponding author. The data are not publicly available due to privacy restrictions.
